# Effects of Game-Based Learning on Piano Music Knowledge Among Elementary School Pupils: Pretest-Posttest Quasi-Experimental Study

**DOI:** 10.2196/80766

**Published:** 2026-01-19

**Authors:** Yun Wang, Wee Hoe Tan, Qiaoling Ye, Tingting Gu

**Affiliations:** 1 Faculty of Social Sciences and Liberal Arts UCSI University Cheras, Kuala Lumpur Malaysia; 2 College of Music and Education Chuzhou University Chuzhou, Anhui China; 3 Chongqing University of Posts and Telecommunications Chongqing, Chongqing China; 4 Chengbei Primary School Chaohu, Anhui China; 5 Faculty of Teacher Education Chaohu University Chaohu, Anhui China

**Keywords:** quality education, gamified learning, piano education, music knowledge, school pupils

## Abstract

**Background:**

Music education is central to holistic child development; yet, traditional piano instruction in China often emphasizes rote memorization at the expense of meaningful understanding. Elementary school pupils, particularly novices, frequently struggle with rhythm, melody, and music theory. Game-based learning (GBL), which applies game elements to teaching, may address these challenges by enhancing engagement, providing immediate feedback, and fostering long-term learning.

**Objective:**

This study aimed to examine the effects of a GBL module for piano education on elementary school pupils’ music knowledge in Anhui Province, China, compared to traditional instruction.

**Methods:**

A quasi-experimental, nonequivalent control group pretest-posttest design was used. Participants were allocated nonrandomly to experimental and control groups based on scheduling feasibility and teacher availability. A total of 60 novice piano learners (mean age 8.8 years, SD 1.16 years; 16 boys and 44 girls) from 3 elementary schools were assigned to either the GBL group (n=30) or the control group (n=30). Music knowledge was measured using the standardized Level 1 Basic Music Written Test before and after an 8-week intervention. Nonparametric analyses were applied, including Mann-Whitney *U*, Wilcoxon signed-rank, and McNemar item-level analyses. Rank-based effect sizes (*r*) and 95% CIs were reported where applicable.

**Results:**

Baseline differences were present, with the control group scoring higher at pretest (median 52, IQR 24-76) than the GBL group (median 28, IQR 16-64; Mann-Whitney *U*=265.50; *r*=–0.35; 95% CI –0.39 to –0.32; *P*=.006). After the intervention, the GBL group significantly outperformed controls (median 100, IQR 88-100 vs median 60, IQR 40-92; Mann-Whitney *U*=4.0; *r*=–0.87; 95% CI –0.90 to –0.83; *P*<.001). Within-group analyses confirmed significant pre-post improvements for both groups (control *Z*=–3.24; *r*=–0.59; *P*=.001; and GBL *Z*=–4.81; *r*=–0.88; *P*<.001). Item-level McNemar tests showed significant improvements (*P*<.05) in 5 of 25 items for the GBL group. Missing data were negligible (<2%) and handled via listwise deletion after Little’s missing completely at random (MCAR) confirmation (*P*=.08).

**Conclusions:**

The GBL module significantly improved pupils’ music knowledge, overcoming baseline disparities and producing posttest score gains with consistent mastery. The innovation of the study lies in the systematic integration of gamification with Orff and Dalcroze pedagogy through the Sidek Module Development Model, which distinguishes it from previous music education studies that examine gamification in isolation. By providing a validated, cost-effective, and scalable instructional module, the study contributes empirical evidence to the field of game-based music education and other practical implications for improving piano instruction in resource-constrained elementary school settings.

## Introduction

Music education is a cornerstone of holistic development for elementary school pupils in China, nurturing not only artistic literacy but also cognitive, emotional, and social growth. The piano is highly regarded among musical instruments due to its versatility and extensive skill set. In recent decades, young pupils have taken up piano, a phenomenon known as the “piano heat” [1]. This increase shows a societal commitment to arts education, as piano skills enhance a child’s cultural capital and allow for creative self-expression. The setting of this study, Anhui Province, has seen piano education grow along with national trends and demonstrate regional commitment to early artistic development. Despite the excitement for piano learning, it remains difficult to ensure that pupils develop a profound, meaningful understanding of music beyond mechanical proficiency.

Traditional piano teaching methods that emphasize rote memorization and mechanical practice typically fail to interest and comprehend young learners [2,3]. Due to their cognitive and emotional growth, elementary school pupils require pedagogical approaches that match their capacities. Their short attention spans and abstract nature make them struggle to grasp rhythm, melody, and basic music theory [4-6]. The lack of musical knowledge might also prevent pupils from connecting emotionally with the subject, reducing their motivation and interest [7]. These obstacles highlight the need for innovative teaching strategies that improve music knowledge and engagement.

Contemporary research identifies critical shortcomings in traditional piano beginner teaching, including a reliance on single-minded evaluation models, a neglect of process-based instruction, and a teaching focus that often prioritizes technical results over genuine artistic development. A key challenge remains the comprehensive cultivation of musical literacy, which extends far beyond the accurate reproduction of notes and rhythms [8]. To address these pedagogical gaps, game-based learning (GBL) has emerged as a promising intervention. GBL represents a fusion of informal and formal education, leveraging the interactive nature of games to deliver structured content. Scholarly reviews confirm that interactive mechanics of GBL, such as the use of challenges, rewards, and progress tracking, provide learners with a sense of autonomy and competence [9]. Furthermore, well-designed educational games can deepen a pupil’s understanding of abstract content, encouraging them to find diverse solutions and train their creative and critical thinking skills. These mechanisms align directly with the goal of developing comprehensive musical literacy that transcends simple rote technique [10].

Gamified learning, which uses game design to boost engagement and learning, is a transformative solution. Reimagining piano exercises as engaging, entertaining experiences makes abstract concepts more approachable and fosters continuous involvement in music instruction [11]. Gamified learning works well in music education with obstacles, rapid feedback, and incentive systems [12]. Gamified learning also fits well with music pedagogy like the Orff and Dalcroze Eurhythmics, which emphasize active participation, rhythm, and movement to meet young learners’ developmental needs [13-16].

In Anhui, elementary school pupils’ piano music knowledge is still lacking despite research showing the benefits of gamified learning in other educational settings and music contexts. Regional specificity is essential for meaningful research since local teaching practices and resource availability may affect gamified intervention efficacy [17]. Gamified music education literature emphasizes short-term outcomes, like skill acquisition, but lacks data on long-term knowledge retention and conceptual understanding [10]. Few studies have examined how gamified learning can be integrated with traditional piano teaching methods or tailored to individual learner differences.

This study examines how a tailored GBL module affects elementary school pupils’ music knowledge in Anhui. Innovative, evidence-based strategies to meet these learners’ unique issues are used to promote music instruction. The design of the GBL module was grounded in established pedagogical principles of Orff-Schulwerk and Dalcroze Eurhythmics, which provided the theoretical foundation for integrating movement, rhythm, and embodied cognition into game tasks. These theories collectively informed the development of a multimodal, developmentally appropriate, and musically grounded GBL intervention for elementary pupils.

The findings should make theoretical and practical contributions to the field. The study expanded on Orff and Dalcroze frameworks to better understand how GBL can promote cognitive, emotional, and creative development in piano teaching. It will provide a validated GBL module for educators to enhance teaching, particularly in resource-constrained or outdated curriculum situations. The findings may also inform policymakers about how gamified learning might improve music education and offer a scalable approach. Accordingly, this study aims to examine the effects of a GBL module on piano music knowledge among elementary school pupils in Anhui province, China, and hypothesizes that pupils receiving the GBL intervention demonstrate greater improvements than those receiving traditional instruction.

## Methods

### Study Design

This study used a quasi-experimental, nonequivalent control group pretest-posttest design to examine the effects of a GBL module for piano education on elementary school pupils’ music knowledge in Anhui Province, China. This design allowed comparisons between an intervention group receiving gamified piano instruction and a control group receiving traditional instruction. The report follows the Transparent Reporting of Evaluations with Nonrandomized Designs (TREND) statement to enhance transparency and reproducibility [18].

### Setting

The study was conducted at a private piano training center located in Anhui Province, China. Three elementary schools partnered with the training center to provide access to novice piano learners. Data collection took place between February and April 2025, with 8 consecutive weekly sessions delivered during this period. Teacher workshops to standardize instructional methods were conducted in January 2025 before the intervention.

### Participants

A total of 60 elementary school pupils aged 7 to 12 years were recruited through a series of briefing sessions held at the training center and affiliated schools. Eligibility criteria included (1) no previous formal piano training, (2) enrollment in the first year of piano lessons at the training center, and (3) parental consent and child assent for participation. Pupils with known hearing impairments or learning disabilities that could substantially affect music learning were excluded. Participants were systematically assigned to an experimental or a control group using a quasi-random sampling procedure: the first 60 pupils whose parents or guardians signed consent forms were enrolled, and every alternate consenting child was assigned to one of the two groups. This resulted in 30 participants in the gamified learning group and 30 in the control group. The final sample comprised 16 boys and 44 girls, consistent with broader trends in higher female participation in music education. Table 1 summarizes demographic characteristics. Although participants were alternately allocated, the procedure did not constitute true randomization. To minimize allocation bias, baseline demographics and pretest scores were compared across groups to assess equivalence.

**Table 1:** Demographic characteristics of elementary school pupils participating in a quasi-experimental study of game-based piano learning in Anhui, China.

Demographic variable			Experimental group, n		Control group, n		Total, n	
**Age (years)**								60
	7	2		4		6		
	8	6		17		23		
	9	7		6		13		
	10	11		3		14		
	11	3		0		3		
	12	1		0		1		
**Sex**								60
	Male	11		5		16		
	Female	19		25		44		
**Year in school**								60
	Year 1	3		9		12		
	Year 2	3		12		15		
	Year 3	13		9		22		
	Year 4	7		0		7		
	Year 5	3		0		3		
	Year 6	1		0		1		

### Intervention

The experimental group received an 8-week GBL module for piano education designed using the Sidek Module Development Model (SMDM). The module consisted of eight 45-minute sessions incorporating music games, including the “Central C Knocking Game,” “Rhythm Clapping Game,” and “Music Notation Song Game.” Content followed a progressive trajectory from basic note recognition to more complex rhythm and score interpretation, guided by Orff and Dalcroze pedagogical principles. All lessons were delivered on an individual basis, with hands-on keyboard practice embedded within structured teacher-led activities. Attendance was recorded weekly, and teachers completed session-by-session fidelity checklists to ensure consistent adherence to the intended instructional sequence for both the gamified and traditional teaching conditions.

The control group received traditional piano instruction over the same period. Both groups were taught by the same 5 piano teachers, who attended a 3-day workshop to ensure standardized delivery of both instructional methods. Teachers applied gamified methods for the intervention group and conventional techniques for the control group. Neither participants nor teachers were blinded due to the nature of the educational intervention; however, outcome assessors scoring the written test were blinded to group allocation.

### GBL Module Development

#### Overview

The SMDM, proposed by Sidek and Jamaludin [19], emphasizes systemic structure and the operational feasibility of instructional activities, making it particularly suitable for developing educational modules. In the context of a GBL module for piano education for elementary school pupils, the SMDM iterative and user-centered approach aligns seamlessly with GBL principles, fostering an engaging and effective learning experience tailored to young learners’ needs. The structured yet flexible model of SMDM supports the creation of a GBL module for piano education by prioritizing user engagement and iterative refinement. The SMDM was used to create the GBL module to enhance elementary school pupils’ piano music knowledge in Anhui [20]. The SMDM is a user-centered instructional design framework that emphasizes comprehensive needs analysis and iterative feedback to create effective multimedia learning tools. The model prioritizes user involvement and adaptability, which are essential for addressing the dynamic challenges in piano education, such as motivation, posture, and skill progression. While the original SMDM framework consists of 2 main stages, the study adapted it into 5 phases (design, development, assembly, validation, and evaluation) to provide a more granular and systematic approach tailored to the development of a GBL module for piano education. This expansion was necessary to accommodate the complexities of integrating technology, gamification, and pedagogical scaffolding in piano learning for elementary school pupils. The needs analysis stage was refined into the design phase for thorough user requirement gathering, while the multimedia development stage was subdivided into development, assembly, and validation phases to ensure iterative refinement and quality assurance. The evaluation phase was added as a distinct step to assess the effectiveness of the module after its implementation, aligning with empirical validation of SMDM. This 5-phase adaptation mirrors the scaffolding process in one-to-one piano lessons, where instruction progresses systematically from foundational to advanced skills, and supports targeted interventions. This systematic approach made the module pedagogically robust, engaging, and customized to young learners. Design, development, assembly, validation, and evaluation built on each other to create a cohesive educational tool.

#### Design Phase

The main goal of the module was to develop a dynamic GBL approach that elevates piano music knowledge for 7- to 12-year-olds. The aim was to craft a challenging but fun learning experience that deepened musical understanding. A game-based piano learning experience for elementary school pupils should be engaging and appealing [21]. The module emphasizes engaging experiences, clear goals, immediate feedback, diverse gaming techniques, and adaptive difficulty levels. Games that reinforce piano music knowledge use these principles. A comprehensive needs analysis identified learning challenges and ensured the module addressed them through targeted gamification. The 4Keys2Fun framework [22] was used to keep pupils engaged by incorporating social interaction, challenge, exploration, and purposeful learning to improve musical knowledge retention.

#### Development Phase

Content and instructional strategies were carefully selected during development to support a progressive learning trajectory. Music was used to teach specific knowledge outcomes, starting with note recognition and rhythm in “Please Play” and advancing to complex structures in “Church Organ” and “Yankee Doodle.” Teacher demonstration, GBL, guided practice, rhythmic chanting, and formative feedback were used to teach piano. “Rhythm Clapping Game” (pupils competed to replicate rhythmic patterns in pairs, turning practice into a lively and collaborative challenge that reinforced knowledge while fostering peer learning), “Musical Notation Song Game,” and “Central C Knocking Game” were embedded to improve rhythm, note positions, and key identification. A piano and copies of John Thomson’s Easiest Piano Course 1 were needed for 45-minute sessions, ensuring the module is accessible. The teacher demonstrated real-time modeling to explain music.

#### Assembly and Draft Completion

A backward design model prioritized piano music knowledge outcomes before aligning assessments, content, and activities [23]. This concentrated and unified the framework. A total of 8 successive lessons with objectives, assessment tools, and resources guided pupils from basic note recognition to sophisticated musical interpretation in the final edition. This systematic assembly ensured that every part improved musical comprehension.

#### Validation and Pilot Testing

Validation was essential for module reliability and suitability. Three music pedagogy specialists critically assessed its content and design, followed by semistructured interviews with 2 piano teachers. Their suggestions improved content alignment and game dynamics. After expert approval, a pilot test with 17 students over 8 weeks (November 19, 2024, to January 14, 2025) provided practical input and revisions to ensure the readiness of the module for broader use. The 17 participants were novice learners aged 7 to 11 (mean age 8.8, SD 1.16) years, with 11 girls and 6 boys, mostly in Year 2 and Year 3 of elementary school.

#### Piano Education GBL Module

This basic school package has 8 lessons that establish musical principles through individual works. It uses games like “Central C Knocking Game,” “Five-Line Speech Practice,” and “Right-Hand Shooting Game” (students aimed to strike correct notes under time pressure, and their excitement translated into improved recognition and accuracy) to enhance learning. The curriculum systematically teaches piano key identification, note values, rests, and specific notes (C, D, B, E, A, G, and F). It emphasizes practical exercises to improve finger independence, rhythmic precision, and technical fluency on the piano. It also promotes curiosity, self-confidence, and music appreciation through interactive games and collaborative activities. Rhythm accuracy, hand coordination, and note and rest recognition are used to assess student progress, ensuring evidence-based musical progression. This structured technique improves musical proficiency and makes learning fun, supporting game-based music education.

### Assessments and Outcomes

The primary outcome was music knowledge, assessed using the Level 1 Basic Music Written Test developed by the Chinese Music Academy. This 25-item multiple-choice test evaluates aural recognition (tones, melodic contours, dynamics, tempo, and rhythmic structures) and theoretical knowledge (pitch, solfège, intervals, meter, and musical symbols). The test was administered at baseline (pretest) and at the end of the intervention (posttest). Exposures included the gamified learning intervention, while potential confounders such as age, grade level, and baseline knowledge were recorded.

### Study Size

The study included 60 participants (30 per group). The sample size was determined pragmatically based on the capacity of the training center and the number of eligible consenting pupils during the recruitment window. Although no formal power calculation was conducted, a sample of 30 per group has been shown in previous quasi-experimental studies to detect medium-to-large effects in educational outcomes.

### Recruitment Procedures

Recruitment was conducted in collaboration with school administrators and the piano training center. Parents and guardians were provided with written study information, and informed consent was obtained prior to enrollment. Children also provided verbal assent. Recruitment was based on voluntary self-selection following briefing sessions conducted at the partner schools and training center. All eligible pupils whose parents provided consent during the 2-week recruitment window were included, and no incentives were offered.

A total of 86 pupils were screened for eligibility; 60 met the inclusion criteria and consented to participate. All 60 pupils were allocated to one of the two study conditions (30 experimental and 30 control). All participants received the assigned intervention, completed the 8 sessions, and completed both pretest and posttest assessments. No participants were lost to follow-up, and no protocol deviations occurred.

### Data Analysis

All analyses were conducted using SPSS (version 29.0; IBM Corp). The normality of the pretest was assessed using the Shapiro-Wilk test, which indicated nonnormal distributions; hence, nonparametric statistical tests were chosen. Mann-Whitney *U* tests were carried out to compare the control and experimental groups in every pretest and posttest. Rank-based effect sizes (*r*) and 95% CIs were measured where applicable. Wilcoxon signed-rank tests examined changes from pretest to posttest within each group. McNemar tests assessed changes in correct responses for individual items on the 25-item test [24]. Data completeness was assessed, in which only less than 2% of responses were missing, and Little’s missing completely at random (MCAR) test indicated data were missing completely at random (*P=*.078). As the proportion was small, listwise deletion was applied. No imputations were performed. Interpretations emphasized both statistical significance and precision of effect estimates, with CI reporting throughout.

### Ethical Considerations

This study was approved by the Institutional Ethics Committee, UCSI University, under approval number IEC-2024-FOSSLA-0167, dated November 19, 2024. Written informed consent was obtained from all legal guardians, and verbal assent was provided by children before participation.

Confidentiality was ensured by anonymizing all datasets and storing them securely with restricted access. No personal identifiers were included in analyses or publications. Participants were not given financial incentives, but small educational gifts (eg, notebooks and stationery) were provided as tokens of appreciation. No identifiable photographs were taken; all supplementary materials were free of personal identifiers. Where identifiable images were unavoidable, written consent was obtained from both pupils and guardians.

## Results

The pretest results showed a baseline piano music knowledge difference between groups (Table 2). The control and experimental groups had considerable piano music knowledge gaps before the intervention. The control group recorded a median pretest score of 52 (range 24-76) and a mean of 47.73 (SD 14.59), while the experimental group had 28 (range 16-64) and 36.13 (SD 12.92). In Table 3, a Mann-Whitney *U* test revealed a statistically significant difference (Mann-Whitney *U*=265.50; *P*=.006), indicating that the control group began with a higher level of knowledge.

**Table 2:** Pretest and posttest piano music knowledge scores among elementary school pupils receiving an 8-week game-based learning module versus traditional piano instruction in Anhui, China.

	Pretest scores			Posttest scores		
	Control^a^	Experimental^b^	Both	Control^a^	Experimental^b^	Both
n	30	30	60	30	30	60
Median (IQR)	52 (32-60)	32 (28-45)	40 (28-56)^c^	60 (51-65)	100 (95-100)	90 (60-100)^d^
Mode	60	28	28	60	100	100
Mean (SD)	47.73 (14.59)	36.13 (12.92)	41.93 (14.86)	58.67 (11.71)	97.33 (3.68)	78 (21.31)
Minimum	24	16	16	40	88	40
Maximum	76	64	76	92	100	100

^a^Control group (n=30).

^b^Experimental group (n=30).

^c^*P*=.006.

^d^*P*<.001.

**Table 3:** Between-group comparison of piano music knowledge at pretest and posttest using Mann-Whitney U tests in a quasi-experimental study of elementary school pupils in Anhui, China.

	Mann-Whitney *U*	*Z*	*r* (95% CI)	*P* value
Pretest	265.50	–2.75	0.35 (–0.39 to –0.32)	.006
Posttest	4.00	–6.71	0.87 (–0.90 to –0.83)	<.001

To evaluate the effectiveness of an 8-week GBL intervention on piano music knowledge, Mann-Whitney *U* tests were conducted to compare pretest and posttest scores between the experimental group (n=30) and the control group (n=30). Descriptive statistics and test results are presented in Table 3. No adverse events or unintended effects were observed in either study condition throughout the 8-week intervention.

In the pretest, a significant difference was observed between the experimental and control groups (Mann-Whitney *U*=265.50; *Z*=–2.75; *P*=.006), with the experimental group demonstrating higher scores compared to the control group. The effect size was medium (*r*=0.35, 95% CI –0.39 to –0.32), indicating a moderate baseline advantage for the experimental group.

In the posttest, the experimental group significantly outperformed the control group (Mann-Whitney *U*=4.00; *Z*=–6.71; *P*<.001). The experimental group achieved a median score of 100 (range 88-100; mean 97.33, SD 3.69), while the control group had a median score of 60 (range 40-92; mean 58.67, SD 11.71). The effect size was large (*r*=0.87; 95% CI –0.90 to –0.83), reflecting a substantial improvement in the performance of the experimental group. These results suggest that the 8-week GBL module effectively reversed the baseline performance patterns, closing the initial knowledge gap and significantly enhancing piano music knowledge in the experimental group compared to the control group.

The posttest scores achieved by the experimental group indicate a high degree of knowledge mastery, with reduced variability, in contrast to the more varied outcomes observed in the control group. The decreased SD (SD 3.69) in the experimental group suggests more consistent learning gains, reinforcing the ability of the GBL module to achieve uniform learning outcomes. Table 4 shows pretest and posttest score disparities demonstrating the effect of the intervention. The experimental group experienced a median gain of 64 points (range 36-84) with a mean improvement of 61.20 (SD 12.61). The pronounced disparity in knowledge gains shows that the GBL module promotes significant learning progress.

**Table 4:** Differences in piano music knowledge scores between pretest and posttest among elementary school pupils receiving game-based versus traditional piano instruction in Anhui, China

	Score difference between pretest and posttest		
	Control group	Experimental group	Control group and experimental group
n	30	30	60
Median (IQR)	8 (4-16)	64 (55-68)	36 (8-64)
Mode	4	64	64
Mean (SD)	10.93 (11.64)	61.20 (12.61)	36.07 (28.05)
Minimum	–4	36	–4
Maximum	64	84	84

To assess the effect of an 8-week GBL intervention on piano music knowledge, the Wilcoxon Signed Ranks test was conducted to compare pretest and posttest scores within the experimental group (n=30) and the control group (n=30). Results are presented in Table 5.

**Table 5:** Within-group comparison of pretest and posttest piano music knowledge using Wilcoxon signed-rank tests among elementary school pupils in Anhui, China.

Group	*Z*	*r* (95% CI)	*P* value
Control group	–3.24	–0.59 (–0.66 to –0.53)	.001
Experimental group	–4.81	–0.88 (–0.94 to –0.82)	<.001

Both groups demonstrated significant improvements in piano music knowledge from pretest to posttest. For the control group (*Z*=–3.24; *P*=.001), a substantial increase was observed (Z=–3.24; *P*=.001), with a large effect size (*r*=–0.59, 95% CI –0.66 to –0.53). The experimental group exhibited a more robust improvement (*Z*=–4.81; *P*<.001), with a larger effect size (*r*=–0.88, 95% CI –0.94 to –0.81). The higher *Z* value and lower *P* value in the experimental group with a larger effect size indicate that the GBL intervention led to greater knowledge growth compared to the control group. These findings, combined with the between-group comparison (see Table 3), suggest that the GBL module significantly enhanced learning outcomes, effectively closing the baseline knowledge gap in the experimental group.

McNemar tests were conducted on pretest and posttest scores for 25 test items to assess significant changes in performance. The item-level McNemar tests were exploratory ancillary analyses intended to examine differential item difficulty and the distributional impact of the intervention across 25 outcomes.

For the experimental group, McNemar tests were applied to each of the 25 piano knowledge items (Table 6). The analysis revealed statistically significant improvements (*P*<.05) in 5 items, highlighting the targeted effectiveness of the GBL module [25]. The remaining 20 items showed no statistically significant changes (*P*>.05).

**Table 6:** Item-level McNemar analyses of changes in piano music knowledge test items following an 8-week game-based learning (GBL) intervention among elementary school pupils in Anhui, China.

	Test items	Counts					Mode		*P* value
				Post^a^					
				W^b^	C^c^				
1	Listen and discern which sound is higher.					W-W		.04	
		Pre^d^	W	13	3				
		—^e^	C	12	2				
2	Listen and discern which sound has a shorter duration.					W-W		≥.99	
		Pre	W	11	6				
		—	C	7	6				
3	Listen and discern, choose the correct pitch.					W-W		.11	
		Pre	W	16	2				
		—	C	8	4				
4	Listen and discern which one is the Concord interval.					W-W		.008	
		Pre	W	21	0				
		—	C	8	1				
5	Comparing melody intervals, which interval is farther away?					C-W		.001	
		Pre	W	10	2				
		—	C	16	2				
6	Listen and discern the direction of melody.					W-W		.27	
		Pre	W	10	4				
		—	C	9	7				
7	Listen and discern the melody, choose the correct beat number.					C-C		.79	
		Pre	W	5	6				
		—	C	8	11				
8	Listen and discern, choose the rhythm within the square.					W-W		N/A^f^	
		Pre	W	16	0				
		—	C	14	0				
9	Listen and discern the melody, choose the correct note within the square.					W-W		.73	
		Pre	W	18	3				
		—	C	5	4				
10	Listen to the melody. What is the style of this piece of music?					W-W; W-C		.80	
		Pre	W	9	9				
		—	C	7	5				
11	Which note has a lower vocal range？					W-W		.79	
		Pre	W	11	8				
		—	C	6	5				
12	Which one is a harmony interval?					W-W		.27	
		Pre	W	13	4				
		—	C	9	4				
13	Choose the correct singing name.					W-C; C-C		N/A	
		Pre	W	0	15				
		—	C	0	15				
14	Choose the correct interval degree.					C-W; C-C		.09	
		Pre	W	7	3				
		—	C	10	10				
15	Which one is the dissonant interval?					C-C		.23	
		Pre	W	9	3				
		—	C	8	10				
16	Which one is the sixteenth rest?					W-C; C-C		N/A	
		Pre	W	0	15				
		—	C	0	15				
17	Choose the correct symbol meaning.					W-C		N/A	
		Pre	W	0	16				
		—	C	0	14				
18	According to the spectrum example, choose the correct beat number.					W-C		<.001	
		Pre	W	1	21				
		—	C	1	7				
19	According to the beat sign, how many bars of the score are shared?					W-C		.01	
		Pre	W	5	12				
		—	C	2	11				
20	Choose the correct sound name and complete the C natural major scale.					C-C		.11	
		Pre	W	3	8				
		—	C	2	17				
21	The instrument in the picture is _______.					C-C		N/A	
		Pre	W	0	12				
		—	C	0	18				
22	The instrument in the picture is _______.					C-C		N/A	
		Pre	W	0	12				
		—	C	0	18				
23	The instruments belonging to the woodwind group are_______.					W-C; C-C		N/A	
		Pre	W	0	15				
		—	C	0	15				
24	Representatives of the “classical music” period.					W-C		N/A	
		Pre	W	0	22				
		—	C	0	8				
25	“Sunset Flute and Drum” belongs to _______.					C-C		NA	
		Pre	W	0	12				
		—	C	0	18				

^a^Post: posttest.

^b^W: wrong.

^c^C: correct.

^d^Pre: pretest.

^e^Not applicable.

^f^N/A: not applicable.

For the control group, McNemar tests were similarly conducted on the 25 items. Five items (#1, #4, #5, #18, and #19) showed statistically significant improvements (*P*<.05), though the gains were less pronounced than in the experimental group. Both groups improved significantly in aural recognition (#1, #4, and #5) and rhythmic analysis (#18 and #19). However, the experimental group showed greater gains in complex tasks like beat identification (#18) and score interpretation (#19), suggesting the GBL module excels at engaging pupils in challenging musical concepts. The improvements in the control group were more stable in simpler tasks (eg, #1 and #4), indicating retention rather than new learning, consistent with the limited scope of traditional approaches. The item-level analysis underscores that the GBL module improves piano music knowledge, particularly in rhythm and score analysis, where the experimental group outperformed the control group. Both groups mastered several things, but the larger and more substantial gains of the experimental group show that the GBL module can enhance music education outcomes.

## Discussion

### Summary of the Main Study

This study examined whether a GBL module for piano education could improve music knowledge among elementary school pupils in Anhui, China, compared with traditional piano instruction. The findings demonstrated that pupils in the gamified learning group significantly outperformed their peers in the control group after the 8-week intervention, achieving near-perfect posttest scores with reduced variability. This suggests that gamification not only facilitated mastery of theoretical and aural skills but also promoted more consistent learning outcomes across participants. The between-group Mann-Whitney *U* test showed a large effect size, while the within-group Wilcoxon analysis confirmed significant pre-post improvements in both groups, with stronger gains in the gamified condition. These results validate the hypothesis of the study that gamified learning would yield superior knowledge acquisition compared to conventional methods, addressing the gap identified in the Introduction. In summary, the evidence confirms that gamification is an effective and scalable pedagogical approach for strengthening music knowledge in early piano education.

A tailored GBL module improved elementary school pupils’ piano music knowledge in Anhui, compared to the control group. Postintervention, GBL module students outperformed the control group by a significant margin. Gamified approaches improve music knowledge, supporting previous research on GBL for music education engagement [11,12]. The near-perfect scores of the experimental group and low variability indicate that the module boosted knowledge acquisition and ensured consistent mastery, a challenge in traditional piano pedagogy.

A comprehensive needs analysis indicated that Anhui elementary pupils struggle with motivation and foundational skills when learning piano. A dynamic, entertaining GBL approach was used to improve piano knowledge, skills, and attitudes. The module promoted musical competency and positive learning habits through gamification principles, including fun, clear objectives, immediate feedback, and adaptability [26]. Addressing these challenges created an engaging experience targeted to young learners’ interests, establishing the groundwork for the research findings.

Starting with “Please Play” to build foundational skills and on to “Church Organ” to refine expressive techniques, the module material was progressive. Structured repetition improved cognitive and motor skills in this sequencing. The instructional design integrated traditional piano pedagogy with gamified activities like the “Right-Hand Shooting Game” and “Five-Line Speech Practice” to improve retention and motivation [27]. These entertaining activities improved rhythm, note recognition, and expression, directly benefiting the understanding of the experimental group.

The lesson design required only a piano and basic multimedia tools, ensuring accessibility across schools in Anhui. Teacher-led demonstrations emphasized real-time feedback and interpersonal connection, which young learners need, above digital media. Tailored feedback allowed pupils to correct errors and refine performance immediately [4], boosting motivation and addressing individual needs [27]. While technology provided aural and visual signals, research suggests that interleaving teacher demonstrations with student imitation is better than audio-only or blocked observation [28]. Thus, the module balanced traditional and technological approaches in a practical, learner-centered environment [28].

### Comparisons to Existing Literature

Quasi-experimental assessments confirmed the efficacy of the module. The pupils improved their piano knowledge and skills from a poor baseline to mastery by the end of the study, outperforming peers in traditional settings. Item-level analysis demonstrated targeted gains in aural recognition (pitch differentiation) and rhythmic skills (beat identification), aligning with the gamified focus [29]. Game-based elements, including challenges, real-time feedback, and rewards, increased the median score of the experimental group much more than the control group. Similar to Molloy et al [21], gamified piano instruction enhanced note accuracy and technical performance compared to traditional techniques, possibly due to greater engagement and skill mastery. Fadhli et al [30] found in a meta-analysis that gamified instruction improves children’s musical knowledge and technical proficiency. These findings support the idea that GBL approaches can significantly improve musical learning outcomes.

Both groups started with similar baselines, but the experimental group improved by posttest. Traditional teaching methods yielded marginal, inconsistent gains in the control group. GBL improves engagement and retention, according to Robert et al [31] and Qian and Jiang [32], who linked it to piano performance. The interactive design of the module addressed the focus of traditional pedagogy on mechanical execution over holistic understanding, resulting in limited development in the control group [33,34].

The success of the experimental group reflects the affective and cognitive benefits of GBL. Gamification increases motivation and self-efficacy, while narrative-driven GBL deepens emotional connections to information [35]. These mechanisms likely drove the experimental group to engage and master. Molero et al [36] observed that gamified systems simplify challenging concepts, which promotes integration of Orff and Dalcroze approaches [15,37]. The focus of the module on aural and rhythmic skills addressed music education gaps, where an outdated curriculum hinders creativity [32]. Beyond immediate outcomes, the performance of the experimental group in analytical tasks (eg, interval comparison) implies GBL fosters higher-order thinking and skill transferability, areas understudied [17,38].

Implementation fidelity was generally high, as teachers followed the structured lesson plans and completed weekly fidelity checklists. However, minor barriers were observed, particularly in maintaining pupil focus during transitions from game activities to reflective discussions. These challenges suggest that future iterations of the module should incorporate more structured debriefing routines to support smoother transitions and deepen conceptual understanding. This study builds on the Orff approach, Dalcroze Eurhythmics, and the SMDM to better understand how gamified learning improves music knowledge in elementary school pupils. The interactive, rhythm-focused exercises of the game-based module increased engagement and musical comprehension, following the Orff approach [13]. The kinesthetic features of the module follow Dalcroze Eurhythmics, which integrates movement and music to improve rhythmic and expressive skills [14], improving pupils’ competency and sensitivity.

Practically, the validated game-based module offers Anhui educators a scalable, resource-efficient tool to modernize piano teaching in often underresourced settings. The experimental group outperformed the control group significantly in knowledge and skills underscores the efficacy of integrating game elements, such as points, levels, and immediate feedback, into instruction [4]. By fostering enjoyment and reducing anxiety, the module also sustains long-term engagement, addressing dropout risks and challenging traditional methods’ focus on technical drills over emotional connection [39,40]. Curriculum designers should thus prioritize holistic, learner-centered approaches.

### Limitations

Despite the promising outcomes of this study, several limitations warrant consideration to contextualize the findings and guide future research. First, the quasi-experimental design used a nonequivalent control group without true random assignment, which may have introduced selection bias, as evidenced by initial differences in pretest scores [24]. Future studies should use true random assignment to enhance internal validity and ensure baseline equivalence between groups. Additionally, the sample size of 60 participants, while sufficient for detecting significant effects, limits the generalizability of findings. The study focused on novice piano learners in a private training center in Anhui, which may not fully represent the diverse educational contexts across China or other regions. Expanding the sample size and including public school settings could strengthen the applicability of the GBL module.

The 8-week intervention period provided valuable insights into short-term knowledge gains but was insufficient to assess long-term retention or the sustained impact of GBL on musical development. Previous research highlights the need for longitudinal data to evaluate deep conceptual understanding and skill retention [10]. Future studies should extend the intervention duration and incorporate follow-up assessments to examine whether the observed gains persist over time. While gamification enhanced engagement, teachers occasionally reported difficulty redirecting pupils’ attention from gameplay to reflective discussion. To mitigate this, future iterations may include structured debrief sessions after each activity.

Another limitation lies in the assessment tool, the Level 1 Basic Music Written Test, which exhibited ceiling effects in the experimental group through their posttest scores. These effects suggest that the test may not have been sufficiently challenging to fully capture knowledge gains, potentially underestimating the impact of the intervention. Moreover, the study primarily measures aural and theoretical knowledge. This focus may overlook other critical aspects of piano learning, such as technical proficiency, emotional expression, or creativity, which are integral to holistic music education [7]. Incorporating multifaceted assessment tools, such as performance evaluations or qualitative measures of student engagement, would provide a more comprehensive understanding of the impact of GBL. Future studies may supplement written tests with recorded piano performances assessed using standardized rubrics, teacher observations, and student portfolios, enabling a more holistic evaluation of musical skill development.

The study did not account for individual learner differences, such as cognitive abilities, learning styles, or previous musical exposure, which may influence the efficacy of GBL [41,42]. The GBL module's one-size-fits-all approach, while effective overall, may not equally benefit all pupils. For instance, item-level analysis revealed variability in performance on complex tasks like interval comparison, suggesting potential confusion for some learners. Future research should explore adaptive GBL designs that tailor content to individual needs. Additionally, the reliance on teacher-led instruction, while resource-efficient, may have introduced variability in implementation fidelity. Although teachers underwent training, differences in their delivery styles could have influenced outcomes.

Finally, this study focuses on a specific context of Anhui, while a strength for regional relevance, but limits its generalizability to other cultural or educational settings. Local teaching practices and resource constraints in Anhui shaped the design of the module, but these may differ elsewhere. Comparative studies across diverse regions could elucidate how contextual factors influence the effectiveness of the GBL module. Furthermore, its integration with traditional pedagogy like Orff and Dalcroze was effective, but its compatibility with other music education frameworks remains underexplored. Investigating hybrid models that combine GBL with varied pedagogical approaches could broaden its applicability.

To address these limitations, researchers should prioritize longitudinal studies with larger, more diverse samples to validate and extend the findings. Randomizing group assignments and incorporating varied assessment methods will enhance the robustness of future investigations. Educators in Anhui and similar regions are encouraged to adopt the validated GBL module, adapting it to local needs while ensuring teacher training emphasizes consistent implementation. Policymakers should consider investing in scalable GBL frameworks, potentially integrating digital tools to enhance accessibility and personalization in resource-constrained settings. By addressing these limitations and implementing these recommendations, the field can further harness the potential of GBL to transform piano education, fostering both musical proficiency and lifelong engagement among young learners.

### Conclusion

The design and validation of the module underscore key implications for music education. Gamification enhanced engagement and knowledge acquisition, suggesting its potential for broader application. Multimodal instruction catered to diverse learning styles, while collaborative practices extended learning beyond the classroom. In conclusion, this GBL module provides a robust, validated approach to early music education for Anhui pupils. By integrating structured content, playful activities, and reflective teaching, it strengthens musical competencies and fosters a positive learning ecosystem, contributing to the evidence base for innovative pedagogy.

This study highlights that integrating gamification into piano pedagogy is both innovative and impactful for early music education. By embedding elements of play, challenge, feedback, and progression into traditional piano lessons, the GBL module transformed abstract musical concepts into engaging learning experiences that fostered consistent mastery among elementary school pupils. This approach is particularly valuable in resource-constrained environments, as the module was designed to be cost-effective, requiring minimal technology beyond a piano and simple teaching aids, while still producing large and meaningful learning gains. The innovation lies in bridging established pedagogical traditions, such as Orff and Dalcroze, with systematic gamification through the SMDM, thereby creating a hybrid framework that supports motivation, knowledge retention, and skill transfer. While traditional methods often emphasize technical drills at the expense of holistic understanding, this study shows that gamification sustains engagement and cultivates deeper conceptual knowledge. Practically, the validated GBL module can be adopted widely by music educators and curriculum designers to modernize piano teaching, reduce dropout risks, and increase accessibility of music learning across diverse school settings. Ultimately, the study demonstrates that gamified learning is not only a pedagogical enhancement but also a scalable and sustainable solution for advancing children’s cultural capital, creativity, and long-term musical engagement.

In conclusion, this study demonstrates that integrating gamification into elementary piano education represents both an innovative and effective pedagogical advancement. The study differs from existing research by systematically embedding GBL within established music education frameworks, namely Orff-Schulwerk and Dalcroze Eurhythmics, and operationalizing this integration through the SMDM. This approach moves beyond previous studies that primarily focus on engagement or short-term skill gains by providing robust empirical evidence of consistent knowledge mastery and reduced performance variability among young learners. The contribution of this study lies in offering a validated, theoretically grounded, and developmentally appropriate GBL module that advances understanding of how gamification can support foundational music knowledge acquisition. From a real-world perspective, the module provides educators and curriculum designers with a scalable, low-cost, and practical solution for modernizing piano instruction, particularly in underresourced educational contexts, while fostering sustained engagement, reducing learner attrition, and strengthening long-term musical development.

## Abbreviations

GBL: game-based learning
MCAR: missing completely at random
SMDM: Sidek Module Development Model
TREND: Transparent Reporting of Evaluations with Nonrandomized Designs

## Data Availability

The data generated and analyzed during this study are not publicly available due to institutional and participant privacy considerations, but are available from the corresponding author upon reasonable request. Deidentified datasets, analysis code, and supplementary materials can be provided to qualified researchers for academic and noncommercial purposes.
